# Electrical stimulation to promote osseointegration of bone anchoring implants: a topical review

**DOI:** 10.1186/s12984-022-01005-7

**Published:** 2022-03-21

**Authors:** Emily Pettersen, Jenna Anderson, Max Ortiz-Catalan

**Affiliations:** 1 Center for Bionics and Pain Research, Mölndal, Sweden; 2 Department of Electrical Engineering, Chalmers University of Technology, Gothenburg, Sweden; 3 Center for Advanced Reconstruction of Extremities (C.A.R.E.), Sahlgrenska University Hospital, Mölndal, Sweden; 4 Department of Orthopaedics, Institute of Clinical Sciences, Sahlgrenska Academy, University of Gothenburg, Gothenburg, Sweden

**Keywords:** Osseointegration, Electrical stimulation, Titanium implants, Bone formation, Prostheses

## Abstract

Electrical stimulation has shown to be a promising approach for promoting osseointegration in bone anchoring implants, where osseointegration defines the biological bonding between the implant surface and bone tissue. Bone-anchored implants are used in the rehabilitation of hearing and limb loss, and extensively in edentulous patients. Inadequate osseointegration is one of the major factors of implant failure that could be prevented by accelerating or enhancing the osseointegration process by artificial means. In this article, we reviewed the efforts to enhance the biofunctionality at the bone-implant interface with electrical stimulation using the implant as an electrode. We reviewed articles describing different electrode configurations, power sources, and waveform-dependent stimulation parameters tested in various in vitro and in vivo models. In total 55 English-language and peer-reviewed publications were identified until April 2020 using PubMed, Google Scholar, and the Chalmers University of Technology Library discovery system using the keywords: osseointegration, electrical stimulation, direct current and titanium implant. Thirteen of those publications were within the scope of this review. We reviewed and compared studies from the last 45 years and found nonuniform protocols with disparities in cell type and animal model, implant location, experimental timeline, implant material, evaluation assays, and type of electrical stimulation. The reporting of stimulation parameters was also found to be inconsistent and incomplete throughout the literature. Studies using in vitro models showed that osteoblasts were sensitive to the magnitude of the electric field and duration of exposure, and such variables similarly affected bone quantity around implants in in vivo investigations. Most studies showed benefits of electrical stimulation in the underlying processes leading to osseointegration, and therefore we found the idea of promoting osseointegration by using electric fields to be supported by the available evidence. However, such an effect has not been demonstrated conclusively nor optimally in humans. We found that optimal stimulation parameters have not been thoroughly investigated and this remains an important step towards the clinical translation of this concept. In addition, there is a need for reporting standards to enable meta-analysis for evidence-based treatments.

## Background

The discovery of osseointegration, the natural phenomenon that defines the process of biological bonding between an implant surface and bone tissue [1], revolutionised the application of limb prostheses by providing the means to skeletal attachment [2] and thus allowing for mechanical coupling and load transfer [3]. Osseointegrated limb prostheses have increased the quality of life for people with amputations by exceeding limitations with conventional socket attachment such as skin irritation and nerve compression, and more recently, allowing for control of the artificial limb and restoration of sensory perception [4, 5]. It has been suggested that in order to obtain a successful osseointegrated prosthesis, the bone-implant interface must be achieved rapidly, be properly maintained, and remain free from infections [6]. Inadequate osseointegration is one of the major factors of implant failure, which in the worst case can lead to implant removal [2]. Non-osseointegrated gaps or pockets represent a risk for bacteria attachment and biofilm formation on the implant surface that can emerge several years after implantation and potentially cause major infections [7]. Another result of failed osseointegration is implant loosening, when the implant is not properly anchored in the bone tissue and thus cannot perform as intended [8]. The osseointegration process can take up to several months depending on the implant design. In situations where early loading of the implant is desired, or when the implant is placed in weakened bone, there is a need to stimulate the osseointegration progression to a rapid and potentially better completion [9, 10].

The concept of enhancing the biological bonding between the implant and bone tissue has been widely investigated including the development and implementation of surface modifications, implant morphology and materials, and surgical implantation techniques [11]. In this article, we analysed and contrasted published studies from the last 45 years that have investigated electrical stimulation modalities as a means to promote osseointegration, with a focus on the electrical stimulation parameters used. Applications using the implant as a stimulating electrode, and titanium as the implant material due to its corrosion resistance and mechanical strength, received specific emphasis during the literature search process. Emphasis was also given to applications that primarily investigated the effect of electrical stimulation and not in combination with another approach, such as mechanical stimuli or surface modifications. English-language peer-reviewed publications were identified until April 2020 primarily in PubMed. Additionally, Google Scholar and the Chalmers University of Technology Library discovery system was used to extend the literature search. Fifty-two publications were identified using the keywords [electrical stimulation + osseointegration], 21 publications [electrical stimulation + osseointegration + titanium implant], and 10 publications [electrical stimulation + osseointegration + direct current]. In addition, two more publications were identified in the references of the reviewed articles, for a total of 55 unique publications. Thirty-eight publications were excluded after reviewing the title and abstract because they did not provide information within the scope of this review. Four out of the remaining 17 publications were excluded after full review, leaving 13 publications covering the scope of this review.

## Promoting osseointegration with electrical stimulation

The use of electrical stimulation to promote osseointegration has been explored over the past decades in both in vitro and in vivo models, with various approaches ranging from different electrode configurations and parameters to sources of electrical current. In vivo research has been exclusively conducted in animal models and not yet reported on human subjects. Electrically stimulated bone growth was originally described by Fukada et al*.* [12] for enhancement of osteogenesis in fracture healing, where the authors suggested that electrical fields were generated by mechanical stress on bone. In turn, these forces compress the tubular structure of bone and cause fluid flow containing ions through the canalicular system, which stimulated bone healing. In other terms, when a force is applied to bone tissue, electrical signals are generated due to the flow of ions and such signalling can be described by the piezoelectric theory [10]. Electrical stimulation has been successfully applied to promote bone formation (osteogenesis) in bone repair applications including non-union bone fractures, osteoporosis, and osteonecrosis [13–16]. In these applications the electric field can be delivered directly, indirectly (capacitive or inductive couplings), and in combination [17]. In 2019, Ehrensberger et al*.* reviewed the literature to examine how electrochemical stimulation can influence bone tissue and bacteria, including studies that showed electrical stimulation to have a positive effect on osseointegration [6]. However, the underlying mechanisms responsible for such an effect are yet undiscovered and the ideal stimulation parameters with the greatest impact remain undefined [6]. Our review concerns said parameters when applied through stimulation via the implant.

Ehrensberger et al*.* stressed the importance to understand the fundamental differences between using electrical stimulation to promote osteogenesis in bone fracture healing compared to promoting osseointegration in bone-anchored implants. In osteogenesis applications, the current is delivered through cathodic stainless-steel wires which are located near the fracture site to enhance the bone formation from one bone segment to another [6]. This is performed without any prior electrode-bone interface. In osseointegration related applications, the current is delivered through the implant to enhance the biological bonding between the implant surface and the surrounding bone. In this manner, the interference regarding the electrochemical properties and the biological bonding between the implant and the adjacent microenvironment is of highest interest [6].

### Electrode configuration, sources and parameters

There exist few ways to provide an electric field based on the configuration of the electrodes (including the implant) and the electric source when intending to promote osseointegration. In this context, voltage describes the electromotive force that has the capacity to move charged particles across the cell membrane in body tissue [18], and more generally, it is the force needed to drive current through a resistance. To create an electric field over a tissue, a voltage is needed to force current via said tissue through conductive electrodes, positioned to distribute the flow of charged particles over the area where osseointegration is desired [18].

#### Electrode configuration

**Fig. 1:**
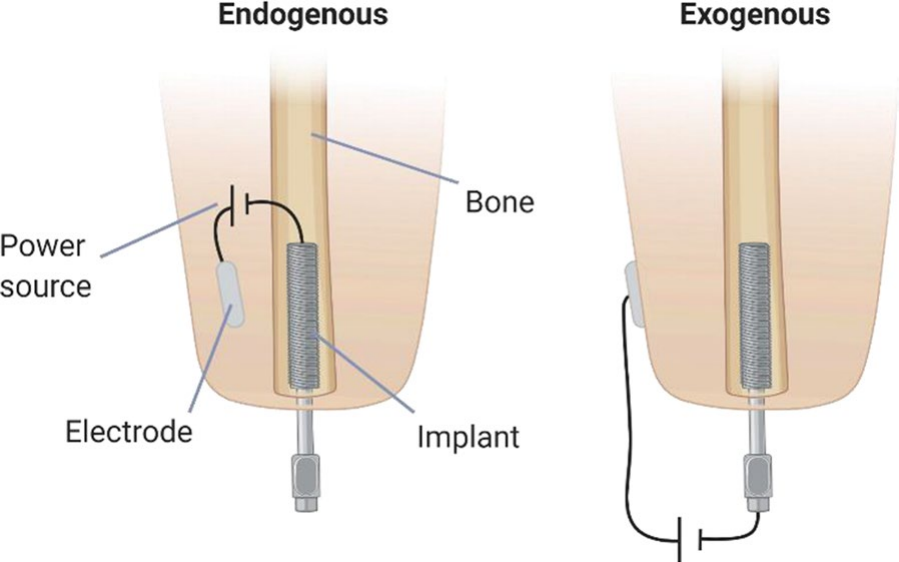
Endogenous vs exogenous electrode configurations for electrical stimulation. Created with BioRender.com

The location of the electrodes impacts the outcome of the electric field and the stimulation can either be endogenous or exogenous. Stimulation with an exogenous configuration uses the implanted fixture and electrodes placed externally on the skin to induce an electric field transcutaneously (Fig. 1). The externally placed electrode can be of different types, for example, ring electrodes which enclose the residual limb [19] or incorporated in an electrical stimulator attached to the implant’s abutment [9]. Endogenous stimulation implies that the electrodes are placed internally, either in the bone or in other soft tissues such as muscles or fat. The majority of in vivo studies have used the implant that is incorporated in the bone tissue as the cathode and the other electrode placed in tissue nearby as the anode [6].

#### Electrical stimulation sources and parameters

**Fig. 2:**
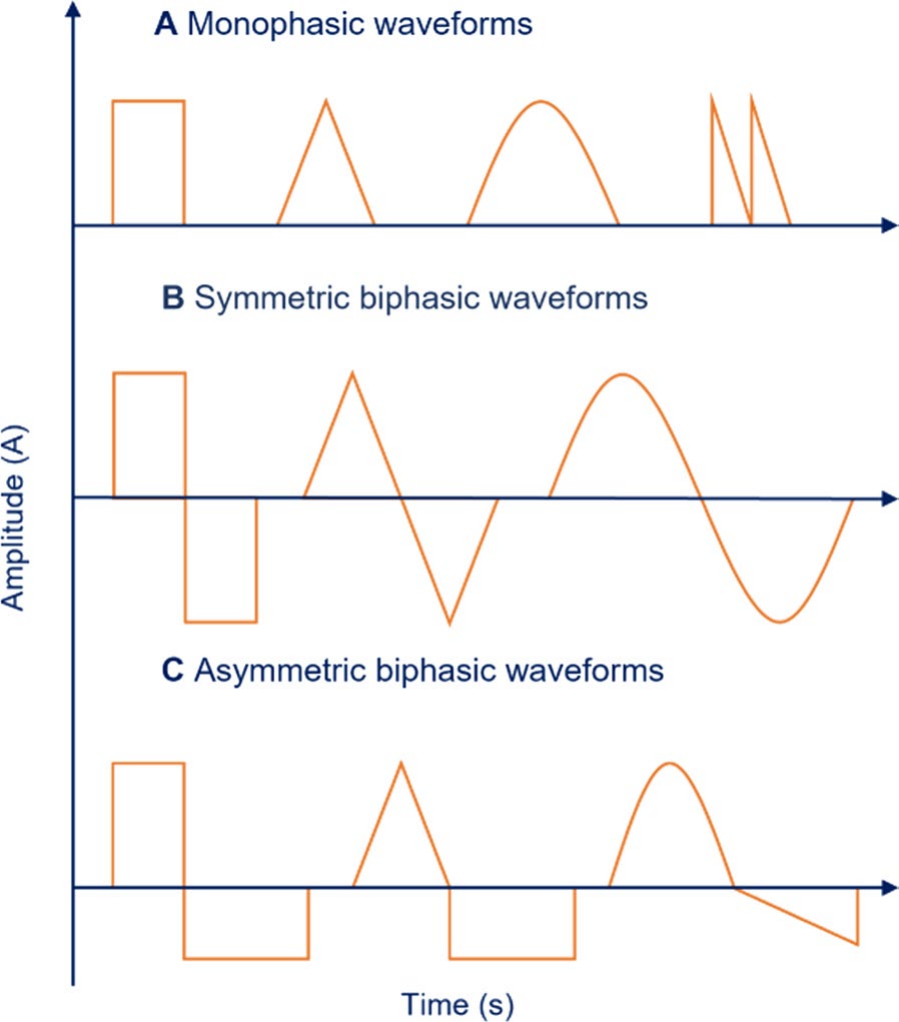
Pulsed electrical current can have many different shapes and waveforms. **A** Examples of monophasic waveforms above the zero baseline. A pulse above the baseline is said to have a positive polarity and a pulse below the baseline is said to have a negative polarity. Examples of biphasic waveforms. The pulse crosses the zero baseline to appear both above and below the baseline. The pulse shape may be **B** symmetric and charged-balanced or **C** asymmetric and/or charged unbalanced

Electrical current defines the rate of flow of charged particles past a specific point and in a specific direction [18]. The flow of current in a wire occurs due to movement of free electrons, whereas the current flow in body tissue arises due to the displacement of ions, such as K^+^, Na^+^ and Cl^−^. An electric current is mostly provided at a steady magnitude and direction (direct current, DC), at a variable magnitude with periodic cycles (alternating current, AC), or in short bursts (pulsed current). AC commonly swings between polarities or directions (positive and negative), but it can also remain within the same polarity while varying its magnitude periodically. Pulsed current is a temporally isolated electrical event where charged particles flow either unidirectionally or bidirectionally [18]. Each event is called a pulse and pulses are separated by periods where there is no flow of current. The frequency of these events is measured in Hertz (Hz) and each lasts a finite amount of time or stimulation period. Pulses can be created in several ways by combining different shapes and waveforms. For example, a pulse can be rectangular with a monophasic waveform above the zero baseline (Fig. 2A). If the pulse is above the baseline it is said to have a positive polarity. Moreover, the pulse can take a biphasic waveform, where the pulse crosses the zero baseline to appear above and below. The pulse shape can either be symmetric (Fig. 2B) or asymmetric (Fig. 2C), and often charged-balanced to maintain the net displacement of charge close to zero after each pulse [20]. In addition to shape, waveform, and polarity, the pulse amplitude (µA), width (µs), frequency (Hz), inter-phase delay (µs), and duty cycle (%) are parameters that need to be defined. Each property or parameter affects the electric field and thus has the potential to cause a different outcome, with potentially negative consequences [20].

### DC stimulation

The most common technique in both in vitro and in vivo applications is invasive DC stimulation. DC stimulation has been shown to have a positive impact on osteoblast functions and to lead to an improved bone-implant interface [6]. Despite beneficial outcomes, there have been some problems associated with the technique. For instance, it can cause accumulation of charged proteins at the electrode surface of opposite charge, which in turn can obstruct current flow at these locations and result in inconsistent current delivery to the cells [21]. Moreover, it has also been reported to trigger formation of reactive oxygen species (ROS) in forms of hydroxyl, hydrogen peroxide, and free radicals [22], and that such ROS can initiate bone resorption [23]. DC stimulation has been suggested to increase pH [21], which causes a toxic environment for cells and tissues, but studies have also reported that this environment can stimulate osteoblastic activity [24–26]. A recently published article by Srirussamee et al*.* reported that the role of faradic by‐products is not dominant in preosteoblast response in terms of bone morphogenetic protein 2 and secreted phosphoprotein 1 mRNA expression, and that the by‐products alone are less effective in promoting bone formation without electrical stimulation. Their results imply that cellular responses from preosteoblasts are predominantly triggered by the mechanism involving electric fields [27]. Overall, the underlying mechanism for the beneficial outcomes of DC stimulation is uncertain and must be further investigated.

### Pulsed current stimulation

Pulsating current was developed in the early 2000s to overcome some of the problems with DC stimulation. One early study investigated the effect of biphasic electrical current pulses (charged-balanced), in order to minimise the net charge accumulation during cell exposure to electrical stimulation [21]. This in vitro study, carried out by Kim et al*.*, showed that biphasic electrical current stimulation induced cell proliferation and vascular endothelial growth factor, a marker for angiogenesis [21]. However, no pH or ROS measurements were performed. This research group has further tested biphasic electrical current stimulation in a canine mandibular model where the chosen parameters in the animal model were based on the in vitro results. They reported a significant increase in bone area and bone-implant contact around stimulated specimens after 3 weeks [9]. Furthermore, our research group pursued an in vitro study where pulsed electrical current was found to exhibit strong positive influence on osteoblast survival, soluble collagen production, attachment and spreading on Ti6Al4V surfaces compared to unstimulated controls [28]. pH measurements revealed no significant difference in pH comparing stimulated specimens and controls [28]. The number of stimulation parameters increases considerably when using pulsating instead of DC stimulation, which needs to be considered when designing the experiments and should be object of further optimisation.

### Assessment of osseointegration

Osseointegration or processes leading to this phenomenon can be assessed by in vitro or in vivo methods. In vitro evaluations can be performed by investigation of osteoblast proliferation, viability, function, and attachment, while in vivo evaluation involves the use of animal models combined with histological assessments and investigations of localised infection or inflammation at the implant site.

The most common assays used in the reviewed in vitro studies are presented in Table 1, where the assays are categorised by the evaluated cell activity, e.g., measurement of metabolic activity, cell proliferation, cell viability, relevant bone markers such as hormones and growth factors, collagen production, cellular attachment, and morphology. These methods were often used in combination, for example, cell proliferation investigated alongside cellular attachment and a relevant bone marker, which can provide a more thorough understanding of the stimulation effect. Numerous evaluation assays are commercially available. Note that one assay can often examine several activities, such as the colorimetric MTT assay that measures metabolic activity via reduction of a yellow tetrazolium salt to purple formazan crystals and thus can be used as an indicator for both cell proliferation and viability [29]. Cell proliferation over a set time period can also be measured by physically removing cells with trypsin-ethylenediaminetetraacetic acid or a lysis buffer, then counting the cells with or without assistance of dye or fluorescent imaging [30]. Hormones and growth factors, such as the bone formation and mineralisation marker alkaline phosphatase, can be analysed by enzyme-linked immunosorbent assay, radioimmunoassay, or quantification of RNA transcript levels in cells using reverse transcription polymerase chain reaction. Qualitative measurements such as scanning electron microscopy (SEM) also enable high magnification imaging for studying cellular attachment and morphology in conjunction with chemical analyses [31].

#### In vitro studies

We found relevant in vitro studies that used a model where cells were cultured directly on electrically-stimulated cathodes to assess acceleration of osseointegration [21, 28, 41–44], (summarised in Table 2). Different stimulation protocols have been investigated in short-term studies lasting between 2 h and 5 days. These studies have shown increased cell growth [21, 43], osteoblastic differentiation [41, 42], soluble collagen production [28] and osteoblast attachment [43] on stimulated surfaces compared to non-stimulated surfaces. However, results have also shown reduction in number of viable cells as well as changed morphology after 24 h of electrical stimulation [44].

About half of the identified studies used current-controlled stimulation, and the other half utilised voltage-controlled sources (constant and alternating). Sivan et al*.* (2013) provided constant catholically polarised implants of 300, 350, 400, 450, 500, 600, 1000 mV with MC3T3-E1 cells cultured directly at the titanium (Ti6Al4V) surface. Their results showed that cell viability and morphology were both time- and voltage-dependent; cells transitioned from viable to nonviable after 10 h at − 400 mV, 6 h at − 500 mV and 2 h at − 600 and − 1000 mV, and 24 h at − 400 mV was detected as a threshold limit for cell apoptosis [44]. Furthermore, Gittens et al*.* (2013) studied differentiation of MG63 cells at catholically polarised surfaces and found a higher rate of differentiation when stimulation was increased from − 100 mV to − 500 mV in steps of 100 mV [42]. Moreover, Dauben et al*.* developed a novel in vitro system where human primary osteoblasts (hPOB) were exposed to voltage-controlled sinusoidal stimulation of 0.2 and 1.4 V_RMS_, frequency of 20 Hz, and stimulation periods of 3 × 45 min per day with a break of 225 min between each stimulation, for 3 days in total [41]. Cells remained viable after the alternating stimulation, but metabolic activity was not significantly higher in stimulated groups compared to controls. However, gene expression showed moderately higher transcript abundance of alkaline phosphatase, collagen type 1 and osteocalcin after stimulation of 0.2 V_RMS_ compared to controls, and enhanced transcript levels of osteocalcin after application of 1.4 V_RMS_ [41]. This study provides an example of how the use of different evaluation methods provides a greater understanding of the effect of stimulation.

Current-controlled stimulation has been primarily investigated in DC and pulses (studies summarised in Table 2). Bodhak et al*.* stimulated human foetal osteoblasts (hFOB) with constant DC stimulation of 5, 15, and 25 μA, respectively, for 15 min every 8 h [43]. After 5 days of stimulation, there was a significant increase in cell-material interaction and density of viable cells in stimulated compared to non-stimulated surfaces. There was also a significant increase between the different stimulation conditions, where 25 μA was more favourable compared to 5 and 15 μA [43]. Furthermore, Kim et al*.* (2006) stimulated rat calvarial osteoblasts (rcOB) with biphasic pulses of 20 μA, (1.5 µA/cm^2^), pulse width of 32 µs and frequency of 3000 Hz. They studied two different stimulation modes: interrupted (6 h daily) and continuous (24 h daily). A significant increase of cell proliferation was found after 2 days with continuous stimulation compared to interrupted stimulation and non-stimulated surfaces. Biphasic stimulation was also found to increase vascular endothelial growth factor production, but did not stimulate osteoblast differentiation [21]. Moreover, in our recent in vitro study [28] MC3T3-E1 preosteoblasts were cultured on Ti6Al4V surfaces and stimulated in a continuous mode for 3 days with current-controlled pulsed electrical stimulation with similar properties as used in peripheral nerve stimulation to restore sensory feedback in artificial limbs [4, 45]. The stimulation was delivered in different conditions where various pulse amplitudes (10 and 20 µA) and frequencies (50 and 100 Hz) were compared. The other parameters were set to a fixed value throughout the experiments; negative pulse width (500 μs), inter-phase delay (50 μs) and sample frequency (100 kSPS). Electrical stimulation, in all different stimulation conditions, was found to have a strong positive influence on osteoblast survival, soluble collagen production, attachment and spreading on Ti6Al4V surfaces compared to unstimulated specimens. Among all test conditions, 20 μA indicated as the most beneficial amplitude, although not significantly higher compared to 10 μA. 100 Hz was found to favour cell proliferation and collagen production compared to 50 Hz and the control. The highest osteoblast density was measured at 20 μA and 100 Hz after 3 days where cells grew almost 120% higher in number and 5 times more collagen production as compared to non-stimulated surfaces. No morphologic or pH difference was found between the stimulated specimens and the control [28].

Whilst the reported outcomes in the in vitro applications seemingly vary, there are several aspects to emphasise. The in vitro studies did not utilise uniform models, where differences in cell type and species (MG63, MC3T3-E1, hPOB, hFOB, rcOB), experimental timeline (stimulation durations andevaluation time points), implant material (titanium, gold), evaluation assays, and stimulation protocols (magnitude, pattern, duration, control unit) may all have contributed to the disparity in reported results. However, despite differences in the models used, various stimulation parameters seem to have a vital role for the enhancement of osseointegration. Studies have shown that osteoblast functions are impacted by the magnitude of the electric field [28, 41–44], stimulation duration [21], frequency [28] and treatment duration [44], while other potentially important parameters such as pulse shape, duty cycle and pulse width are not comprehensively studied.

Most studies failed to report their motivation behind the selection of stimulation parameters. For instance, Bodhak et al*.* stimulated for 15 min every 8 h [43], whereas Dauben et al*.* stimulated for 3 × 45 min daily with a break of 225 min between each stimulation [41]. It is unclear whether the stimulation patterns were selected for scientific reasons, for example, if one pattern was shown in feasibility studies to be more beneficial than another for promoting osteoblast function, or if they were selected for practical reasons, such as limitations of available stimulation hardware.

#### In vivo studies

Electrical stimulation applied directly through the implant to accelerate osseointegration has also been investigated in vivo (summary in Table 3)*,* where several studies have reported promising results [9, 46–50]. Both short (1 day [50]) and long term (up to 12 weeks [10]) stimulation durations have been investigated. Most studies applied a current-controlled stimulation with a magnitude between 5 and 50 μA. The animal models included rabbits, dogs, and sheep, and the titanium implant (Ti6Al4V or commercially pure grade IV) was located in the tibia, mandible, and femur (Table 3). Evaluations of these in vivo studies differed from those used in in vitro studies. Primarily bone quality (porosity, density, bone mineral content including calcium content), bone growth at the implant site (including growth rate, necrosis, and histological assessment of mature or immature bone formation), and degree of skeletal attachment (including histological or SEM assessments such as appositional bone index, bone contact area, and bone-implant contact, as well as mechanical testing) have been assessed.

Isaacson et al*.* applied an electric field with a potential difference of 0.55 V in a rabbit model [47]. The gold coated Ti6Al4V implant was catholically stimulated and placed inside the medullary channel in the femur, and the anode was placed ~ 1.5 cm from the periosteum in the adjacent musculature. Stimulation was ongoing for 3 and 6 weeks. Histological assessments of appositional bone index and mineral apposition rates were not found to be improved by electrical stimulation, nevertheless, stimulation induced trabecular bone growth around the stimulated implants [47].

Previously in a rabbit model, Buch et al*.* applied a constant DC of 5, 20 and 50 μA for 3 weeks [46]. The implant was placed in between the titanium cathode and platinum-iridium anode in the proximal part of the tibial metaphysis. Bone mineral content was significantly higher in 5 and 20 μA specimens compared to 50 μA and controls. However, no qualitative differences between the stimulated groups and the control were found [46]. Infection was not observed, and severe inflammatory reaction was absent in all samples, although a black ring was noted around the anodes in the 50 μA group. In all stimulated samples, bone tissue overgrowth of the cathode was noticed [46].

In a beagle dog model, Bins-Ely et al*.* placed commercially pure titanium grade IV dental implants 2 mm below the crestal bone in the tibia [48]. They applied constant current of 10 and 20 μA for 7 and 15 days, respectively, using an electronic device which was linked to the implant connection area [48]. Their result showed significantly higher bone-implant contact after 15 days of stimulation of 20 μA compared to stimulation of 10 μA and control. However, no significant change in bone-implant contact was observed among the groups after 7 days.

Shayesteh et al*.* applied electrical stimulation of 20 μA between two titanium dental implants placed in the mandibular of a mongrel dog [49]. The stimulation was ongoing for 30 days and the implants were evaluated 90 days post-surgery. The authors reported that the bone contact ratio and local bone formation around the stimulated implants were greater compared to non-stimulated surfaces, but they did not declare if the evaluated implants were cathodes, anodes or both [49].

In a previous mongrel dog model, Colella et al*.* applied constant DC of 15 μA for 1–8 days in porous titanium cylindrical implants inserted in the mid-diaphysis of the femur [50]. Evaluation was made 1-, 2-, and 3-weeks post-surgery where a substantially greater maximum shear stress was required to push out the stimulated implants compared to the controls. However, no qualitative difference was detected for the bone ingrowth. Nevertheless, their result seems to imply that electrical stimulation enhanced bone rate and quantity of bone ingrowth since the stimulated implant appeared to adhere more closely to bone than the control [50].

Dergin et al*.* used a sheep model where they stimulated titanium dental implants placed in the tibia with constant DC current of 7.5 μA for 4, 8, and 12 weeks [10]. The stimulation was ongoing for 12 h per day, 6 h on and 6 h off. No significant increase in bone-implant contact ratio, osteoblast activity, or new bone formation was shown in the stimulated implants compared to the control. Furthermore, biphasic stimulation has also been investigated in a beagle dog model by Song et al. They stimulated titanium dental implants inserted in the mandible with an amplitude of 20 µA/cm^2^, pulse width of 125 µs and a frequency of 100 Hz. The stimulation was ongoing for 7 days and the evaluation was performed 3- and 5-weeks post-surgery. They reported significant increase in newly formed bone area after 3 and 5 weeks compared to controls, but only significant increase in bone-implant contact stimulated specimens after 3 weeks and no increase between stimulated and controls after 5 weeks [9].

The reviewed in vivo applications revealed more consistent results across studies than the in vitro studies. However, it is important to emphasise differences between the study designs, such as the animal model used (rabbit, beagle dog, mongrel dog, sheep), location of the implant (medullary channel, tibia, mandible, femur), metal alloy (commercially pure titanium (grade I–IV) and Ti6Al4V), implant type (dental implant, porous cylinder, cylinder with chambers), experimental timeline (stimulation duration and evaluation time points) and applied electrical stimulation (magnitude, pattern, duration, control unit). Despite the protocol differences among in vivo studies, there are some recurrent similarities. Both Bins-Ely et al*.* and Buch et al*.* investigated different magnitudes of the applied current, 10 and 20 μA and 5, 20, and 50 μA, respectively. Their results showed that there was a clear difference in the outcomes between the applied current, where 10 μA seemed to be too low [48] and 50 μA too high [46] for promoting osseointegration. The observation of a black ring around the anodes by Buch et al*.* is an indication of cell necrosis, which in turn indicates that it is possible to stimulate with a current magnitude that is detrimental. This is consistent with the in vitro studies [28, 41–44] that show a charge-dependent property for cell viability. Bins-Ely et al*.* also suggested that stimulation duration may be an important factor, where no differences between stimulated groups and controls are shown after 7 days, but after 15 days a significant difference was observed in bone-implant contact between 20 μA compared to the controls.

**Fig. 3:**
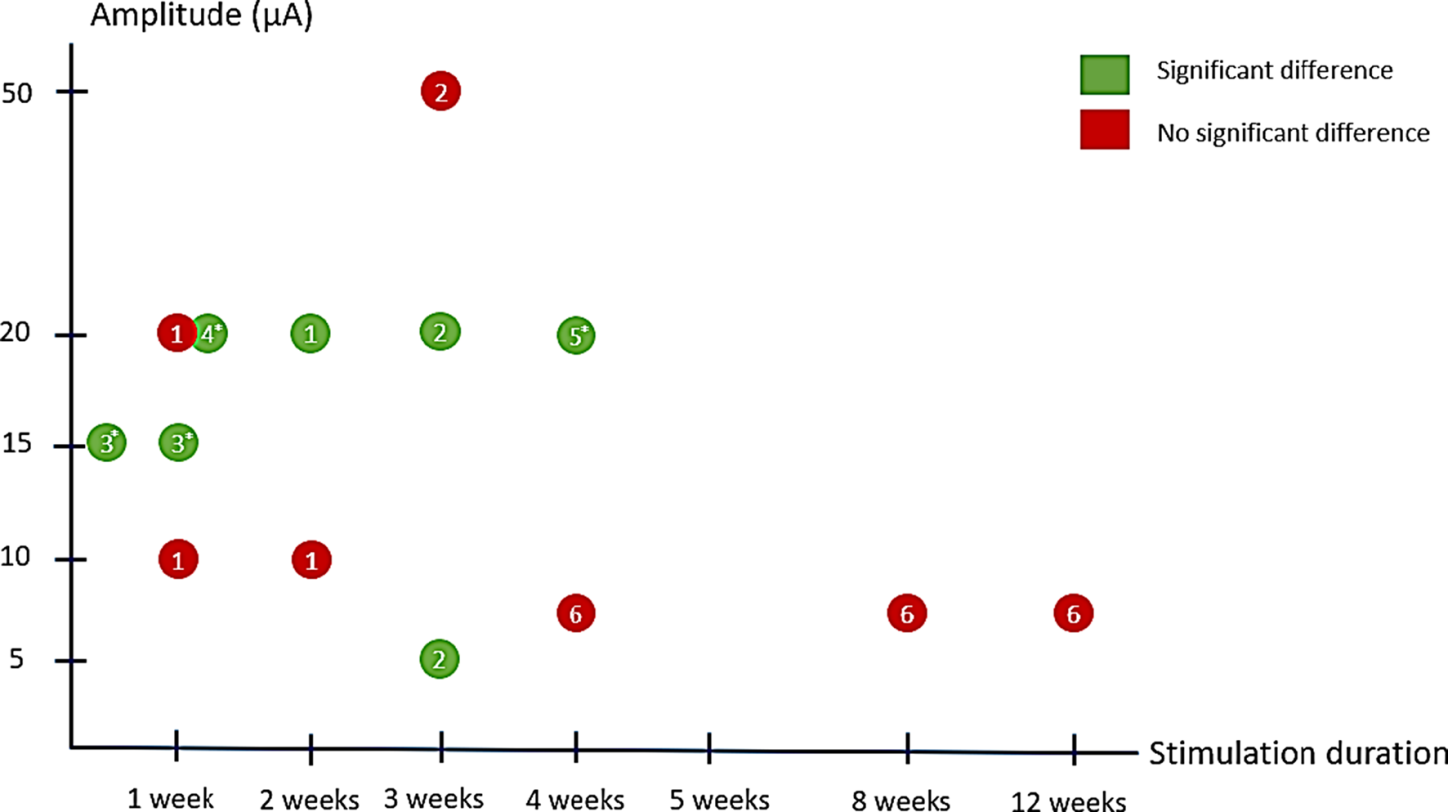
Amplitude vs stimulation duration. Current controlled in vivo studies 1) Bins-Ely et al. (2017) [48] 2) Buch et al. (1984) [46], 3) Colella et al. (1981) [50], 4) Song et al. (2009) [9] 5) Shayesteh et al. (2007) [49], 6) Dergin et al. (2013) [10]. * = Stimulation duration and evaluation assessment time point(s) differ

Studies that performed qualitative analysis of bone of stimulated versus non-stimulated groups did not observe significant differences [46, 50], but bone quantity in terms of bone-implant contact, bone area and bone contact ratio was shown to be significantly greater in the majority of the in vivo applications [21, 46–50]. When plotting the amplitude against the stimulation duration (Fig. 3), the majority of studies with a significant beneficial outcome can be found in the range between an amplitude of 15–20 μA and stimulation duration between 2 and 5 weeks. Note that bone quantity variables were considered for significant difference and the in vivo studies plotted in Fig. 3 used a current-controlled stimulation. Worthy of notice is that increased bone quantity was not observed in the Dergin et al*.* sheep model. When comparing Dergin’s study to the in vivo applications with statistically significant results, there are three main differences in stimulation parameters, namely: current amplitude, stimulation duration, and stimulation protocol. The applied current in the sheep study, 7.5 μA, is below the current limit that previously showed to be too low [48]. The stimulation started directly after implantation and continued until the evaluation assessments which were performed 4-, 8-, and 12 weeks post-surgery which is in the late-phase of implant healing; other studies evaluated at an earlier phase [9, 46, 48–50] (Fig. 3). Dergin’s study also stands out when it comes to stimulation protocol; the current was delivered 12 h per day in a pattern of 6 h on and 6 h off, while the other studies delivered a continuous stimulation through the experiment.

## Conclusion

The aim of this review was to analyse, and contrast published studies that investigated electrical stimulation as a means to promote osseointegration, with a focus on the stimulation parameters and evaluation methods. A total of 13 papers were identified that explored in vitro or in vivo methods for evaluating the potential effect of electrical stimulation on osseointegration. These studies utilised a variety of assessments, including commercially available assays that evaluate cell function, viability, and attachment, as well as histology at the site of implant in animal models. Taken together, both in vitro and in vivo models showed that osteoblasts or bone tissue were shown to be sensitive to the electric field magnitude, stimulation duration, frequency and the protocol used to deliver the stimulation (duty cycle).

We found inconsistencies on stimulation parameters between in vitro and in vivo studies. For example, in vitro studies reported better outcomes following stimulation with amplitudes of 15–25 μA, and yet in vivo studies published after these results were known utilised amplitudes lower than the identified minimum thresholds in vitro (e.g., Dergin et al*.* (2013)). Indeed, it can be difficult to directly translate in vitro methods to the in vivo setting. The in vitro models reviewed here were based on cell culture of one cell line per study. These models are unable to account for bone remodelling (interplay between osteoblast and osteoclast), responses from inflammatory cells and the immune system, and the role of mechanical stimuli. Nevertheless, for the purpose of evaluating the impact of stimulation parameters, in vitro models can serve as an initial phase to reduce the unnecessary use of animals. It may be possible to use more complex cell culture models, such as 3D cell cultures [51] or “organ-on-a-chip” set-ups [52] to better capture 3D interactions in an in vitro setting. This review has revealed the need for further research for the optimisation of stimulation parameters, and equally important, the need for reporting standards when conducting such research, a need that has been already identified in other applications of electrical stimulation in medicine [20, 53].

**Table 1:** In vitro assessment of osseointegration

	Assessment	Assessment description	Outcome measures				
			*Cell proliferation*	*Cell viability*	*Cell adhesion*	*Cell morphology*	Bone markers*
*Colorimetric assay*	MTT^1^	MTT is reduced into fluorescent purple formazan crystals by living cells, which determines mitochondrial activity with a spectrophotometer [32]	X	X			
	WST-1^2^	WST-1 is cleaved to soluble fluorescent formazan by a complex cellular mechanism by living cells, which determines living cell activity with a spectrophotometer [32, 33]	X	X			
*Staining*	LIVE/DEAD	Fluorescent staining of cells using calcein-AM (viable cells), propidium iodide (dead cells) and Hoechst 33,342 (total cells) [34]		X			
	Trypan blue	Staining elimination test where viable cells do not take up the dye, but dead cells are permeable to it [34]		X			
*Imaging*	SEM^3^	Electron microscope that enables high-resolution imaging and generates specimen images by scanning of the surface using a focused beam of electrodes [31]			X	X	
	Fluorescence microscopy	Optical imaging method used to study cell physiology by using fluorescence [35]			X	X	
	CLSM^4^	Optical imaging method used for enhancing optical resolution and contrasting a micrograph by usage of a spatial pinhole to stop out-of-focus light in image formation [36]				X	
*PCR methods*	RT-PCR^6^	A variation of the standard PCR^5^ method where cDNA^7^ is made from RNA^8^ via reverse transcription, which allows amplification of specific mRNA transcripts from small biological specimens [37]					X
	qPCR^9^	Another variation of standard PCR where two elements are added to the standard procedure: fluorescent dye and fluorometer. Widely used for quantifying RNA transcript levels in cells and tissues [37]					X
*Immunoassays*	ELISA^10^	An immunoassay used for quantification which utilises an antibody labelled with an enzyme marker where either the enzyme or antibody is bound to an immunosorbent substrate. The change in enzyme activity is a result of the enzyme-antibody-antigen reaction which is proportional to the antigen concentration [38]					X
	RIA^11^	An immunoassay for quantification of antigen–antibody reaction by usage of a radioactively labelled substance either directly or indirectly to quantify the binding of the unlabelled substance to a specific antibody [39]					X
	Western blotting	Identification assay used to identify proteins or peptides that have been electrophoretically separated via blot relocating from electrophoresis gel into strips of nitrocellulose paper followed by labelling with antibody probes [40]					X

*Common bone markers include alkaline phosphatase (ALP), bone morphogenetic protein 2 (BMP2), collagen type 1 (Col 1), procollagen type 1, osteoprotegerin (OPG), osteocalcin (OC), and vascular endothelial growth factor (VEGF)

^1^MTT, 3-(4,5-dimethylthiazol-2-yl)-2,5-diphenyltetrazolium bromide; ^2^WST-1, 2-(4-Iodophenyl)-3-(4-nitrophenyl)-5-(2,4-disulfophenyl)-2H-tetrazolium; ^3^SEM, scanning electron microscopy; ^4^CLSM, confocal laser scanning microscopy; ^5^PCR, polymerase chain reaction; ^6^RT-PCR, reverse transcription PCR; ^7^cDNA, complementary DNA; ^8^RNA, ribonucleic acid; ^9^qPCR, quantitative PCR; ^10^ELISA, enzyme-linked immunosorbent assay; ^11^RIA, radioimmunoassay

**Table 2:** In vitro studies categorised by cell type, cathode material, evaluation, stimulation parameters, stimulation duration and results

Reference	Cell type	Cathode material	Evaluation	Stimulation parameters	Stimulation duration	Results
Dauben et al*.* 2016 [41]	Human primary osteoblast	Ti6Al4V	WST-1^1^, LIVE/DEAD staining, RT-PCR^2^ (Col 1^3^, ALP^4^, OC^5^), ELISA^6^ (procollagen type 1)	0.2 and 1.4 V_RMS_, frequency of 20 Hz, sinusoidal signal was applied with stimulation periods of 3 × 45 min per day with 225 min break between simulations	3 days	Cells were viable and the metabolic activity was not significantly higher in stimulated groups compared to controls. Gene expression showed moderately higher transcript abundance of Col 1, ALP, and OC after electrical stimulation with 0.2 V_RMS_ compared to controls. Application of 1.4 V_RMS_ resulted in slightly enhanced OC transcript levels while Col 1 and ALP remained unchanged
Gittens et al*.* 2013 [42]	Osteoblast (MG63)	Unalloyed titanium, Grade 2 (ASTM F67)	Trypsin, radioimmunoassay (OC), ELISA (OPG^7^, VEGF^8^)	Anode polarisation of 100 mV and cathode polarisation of 100, 200, 300, 400 and 500 mV	2 h	MG63 differentiation and local factor production was enhanced on catholically polarised surfaces. The effect of the applied electrical polarisation was voltage dependent, with higher potentials promoting a greater osteoblast differentiation
Bodhak et al*.* 2012 [43]	Human foetal osteoblast (hFOB 1.19)	99.7% pure titanium, Grade 2	MTT^9^, SEM^10^, fluorescent staining & CLSM^11^ (vinculin expression)	5, 15, 25 µA constant stimulation for 15 min every 8 h	5 days	Enhanced bone cell–material interactions with increasing amount of DC^12^ stimulation from 5 μA to 25 μA. The highest viable osteoblast cell density was measured on 25 μA stimulated titanium surfaces where cells grew almost 30% higher in number as compared to non-stimulated titanium surface
Sivan et al*.* 2013 [44]	Preosteoblast (MC3T3-E1)	Ti6Al4V	SEM, LIVE/DEAD staining	Cathode polarisation of 300, 350, 400, 450, 500, 600, 1000 mV (vs Ag/AgCl)	24 h	Cell death at commercially pure titanium is both dependent in cathodic voltage and time. Cell culture above − 300 mV showed almost no loss in viability, whereas 100% of the cells were killed at − 600 mV after 24 h
Kim et al*.* 2006 [21]	Rat calvarial osteoblast	Gold	Trypan blue staining, RT-PCR and qPCR^13^, ELISA (VEGF, Bmp2^14^), Western blotting (HIF-1α^15^)	BEC^16^ stimulation with pulse amplitude of 20 µA (1.5 µA/cm^2^), pulse width 32 µs and frequency of 3000 Hz in the interrupted (6 h daily) and continuous mode (24 h daily)	2, 4 and 5 days	Significant increase in cell proliferation after 2 days with stimulation of continuous mode compared to interrupted mode and non-stimulated groups. BEC stimulation increased VEGF production, but did not stimulate differentiation
Pettersen et al*.* 2021 [28]	Preosteoblast (MC3T3-E1)	Ti6Al4V	Physical removal of cells and counted via fluorescence imaging, soluble collagen production through absorbance measurements, SEM, pH measurements	BEC stimulation with pulse amplitudes of 10 and 20 µA, frequencies 50 and 100 Hz, pulse width 500 μs, inter-phase delay 50 μs, sample frequency 100 kSPS, continuous stimulation (24 h daily)	3 days	Stimulation exhibited strong positive influence on osteoblast proliferation, collagen production and spreading on TiAl4V surfaces. 20 μA indicated as the most beneficial amplitude, although not significantly higher compared to 10 μA. 100 Hz was found to favour cell proliferation and collagen production compared to 50 Hz and the control. No morphologic or pH difference was found among the stimulated specimens and the control

^1^WST-1, 2-(4-Iodophenyl)-3-(4-nitrophenyl)-5-(2,4-disulfophenyl)-2H-tetrazolium; ^2^RT-PCR, reverse transcription PCR; ^3^Col 1, collagen type 1; ^4^ALP, alkaline phosphatase; ^5^OC, osteocalcin; ^6^ELISA, enzyme-linked immunosorbent assay; ^7^OPG, osteoprotegerin; ^8^VEGF, vascular endothelial growth factor; ^9^MTT, 3-(4,5-dimethylthiazol-2-yl)-2,5-diphenyltetrazolium bromide; ^10^SEM, scanning electron microscopy; ^11^CLSM, confocal laser scanning microscopy; ^12^DC, direct current; ^13^qPCR, quantitative PCR; ^14^BMP2, bone morphogenetic protein 2; ^15^HIF-1 α, hypoxia-inducible factor 1-alpha; ^16^BEC, biphasic electrical current

**Table 3:** In vivo studies categorised in animal model, implant type and material, evaluation, stimulation parameter, stimulation duration and results

References	Animal model (implant site)	Implant type & material	Evaluation	Stimulation parameter	Stimulation duration	Results
Isaacson et al. 2011 [47]	Rabbit (femur, medullary channel)	Dental implant, Ti6Al4V	SEM^1^, histological assessment (bone ingrowth), porosity analysis, dynamic histomorphometry, (mineral apposition rate), biomechanical testing (degree of skeletal attachment was tested with push-out tests)	0.55 V (1.2 V/cm and 1.82 mA/cm^2^)	3 and 6 weeks	Significant increase of trabecular bone around the implant in the stimulated group compared to non-stimulated. Slightly higher values for appositional bone index and mineral apposition rates in stimulated groups, although no significant values
Buch et al. 1984 [46]	Rabbit (tibial metaphysis)	Cylinder with two chambers, titanium	Histology (qualitative analysis), microradiography followed by a computer-aided density analysis	5 µA, 20 µA and 50 µA constant DC	3 weeks	Significant difference in BMC^4^ with stimulation of 5 and 20 µA. No significant difference of BMC with stimulation of 50 µA and no significant difference in qualitative analysis between stimulated and non-stimulated groups. The cathode was always overgrown with bone tissue in those cases when it had been connected to the simulator
Bins-Ely et al. 2017 [48]	Beagle dogs (tibia)	Dental implant, commercially pure titanium grade IV	BIC^2^ by histology and histomorphometry analysis	10 µA and 20 µA constant DC^3^	7 and 15 days	Significant increase in BIC after 15 days of stimulation of 20 µA compared to stimulation of 10 µA and control group. No significant results between groups after 7 days
Shayesteh et al. 2007 [49]	Mongrel dogs (mandible)	Dental implant, titanium	BCA^5^ and BCR^6^ by histological evaluation, quantitative and qualitative analysis	20 µA, 3 V, constant DC	30 days (evaluation after 90 days)	Significant increase in BCR and local bone formation around the stimulated implants as compared to non-stimulated control implants when evaluated at 90 days
Colella et al. 1981 [50]	Mongrel dogs (femur)	Porous cylinder, titanium	SEM (bone-implant interference), EDAX^7^ analysis (determine the calcium content within the implants), push-out-test (mechanical testing)	15 μA constant DC	1, 6, 7, 8 days (evaluation after 1, 2 and 3 weeks)	A substantially greater maximum shear stress was needed to push out the stimulated implant as compared to the control. No qualitative difference was detected in bone ingrowth between the experimental and control implants. The results imply that ES^8^ promote both rate and quantity of bone ingrowth, since stimulated implant did appear to adhere more closely to bone
Dergin et al. 2013 [10]	Sheep (tibia)	Dental implant, titanium	BIC, degree of osteoblast activity, necrosis, immature bone, and mature bone formation by histologic and histomorphometry analysis	7.5 μA constant DC during a period of 12 h per day (6 h off and 6 h on)	4, 8 and 12 weeks	No significant increase in BIC ratio, osteoblast activity, or new bone formation as compared to non-stimulated controls
Song et al. 2009 [9]	Beagle dogs (mandible)	Dental implant, titanium	BIC and BA^9^ by histological evaluations	BEC stimulation with current density of 20 µA/cm^2^, pulse width of 125 µs and a frequency of 100 Hz	7 days (evaluation after 3 and 5 weeks)	Significant increase in newly formed bone area after 3 and 5 weeks. Significant increase in BIC in specimen after 3 weeks, no significant difference between stimulated and non-stimulated specimens in BIC after 5 weeks

^1^SEM, scanning electron microscopy; ^2^BIC, bone-implant contact; ^3^DC, direct current; ^4^BMC, bone mineral content; ^5^BCA, bone contact area; ^6^BCR, bone contact ratio; ^7^EDAX, energy dispersion analysis by x-rays; ^8^ES, electrical stimulation; ^9^BA, bone area

## Data Availability

Not applicable.

## Abbreviations

AC: Alternating current
DC: Direct current
hFOB: Human foetal osteoblasts
hPOB: Human primary osteoblasts
rcOB: Rat calvarial osteoblasts
ROS: Reactive oxygen species
SEM: Scanning electron microscopy
